# Associations of EDNRA and EDN1 polymorphisms with carotid intima media thickness through interactions with gender, regular exercise, and obesity in subjects in Taiwan: Taichung Community Health Study (TCHS)

**DOI:** 10.7603/s40681-015-0008-7

**Published:** 2015-05-29

**Authors:** Tsai-Chung Li, Chia-Ing Li, Li-Na Liao, Chiu-Shong Liu, Chuan-Wei Yang, Chih-Hsueh Lin, Jen-Hao Hsiao, Chih-Yi Hsiao, Wen-Yuan Lin, Fang-Yang Wu, Cheng-Chieh Lin

**Affiliations:** 1 Graduate Institute of Biostatistics, College of Public Health, China Medical University, 404 Taichung, Taiwan; 2 Department of Healthcare Administration, College of Health Science,, Asia University, 413 Taichung, Taiwan; 3 Department of Medical Research, China Medical University Hospital, 404 Taichung, Taiwan; 4 School of Medicine, College of Medicine, China Medical University, No. 91, Hsueh-Shih Road, 404 Taichung, Taiwan; 5 Department of Public Health, College of Public Health, China Medical University, 404 Taichung, Taiwan; 6 Department of Family Medicine, China Medical University Hospital, 404 Taichung, Taiwan; 7 Ph.D. Program for Aging, College of Medicine, China Medical University, 404 Taichung, Taiwan; 8 Bioinformatics and Biostatistics Core, Center of Genomic Medicine, National Taiwan University, 106 Taipei, Taiwan

**Keywords:** EDNRA;, EDN1;, Polymorphisms;, Intima media thickness

## Abstract

The aim of this study was to evaluate the interacted association between EDNRA and EDN1 polymorphisms and gender, regular exercise, and obesity status on carotid intima media thickness (IMT) in community- dwelling subjects of the Taichung Community Health Study. Five single-nucleotide polymorphisms (SNPs rs1395821, rs1878406, rs5333, rs1800541, and rs5370) of the EDNRA and EDN1 gene were examined in 480 participants from 160 families. The IMT protocol involves scanning the common carotid arteries (CCAs), the carotid bifurcations (bulb), and the origins (first 1 cm) of the internal carotid arteries (ICAs). Generalized linear models with a generalized estimating equation were employed to consider the dependence among family members. After multivariate adjustment, the effects of interactions between EDNRA and EDN1 gene with gender, obesity, and exercise were observed. For gene-gender interaction on CCA IMT, the adjusted mean for men carrying the GA/GG genotype of EDNRASNP rs1878406 was 1.18 times higher than that for men carrying the AA genotype (95% CI: 1.01, 1.37). As for bulb and ICA IMT, the adjusted mean values for women carrying the AC/AA genotype of EDN1 rs5370 was lower than those carrying the CC genotype: 0.89, [0.82, 0.98]; and 0.90 [0.83, 0.99], respectively. We did observe significant effects of EDNRA SNPs rs1395821 and rs5333 in individuals who regularly exercised. A significantly lower adjusted mean in CCA IMT for non-obese individuals carrying EDNRA SNP rs5333 was observed (0.92 [0.86, 0.99]) compared with non-obese individuals carrying the AA genotype. This study first reported significant interactions of EDNRA and EDN1 polymorphisms with gender, regular exercise, and obesity on carotid IMT in Han Chinese participants.

## 1. Introduction

Cardiovascular disease (CVD), including coronary heart disease (CHD), stroke, and peripheral arterial disease, is responsible for a lot of morbidity and premature mortality. The pathogenesis of CVD appears to be multi-factorial. Genetics and environments are two major determinants that result in the development of metabolic syndrome, a cluster of CVD risk factors. These CVD risk factors include high blood pressure, high (pro)-insulin concentrations, excess body weight with central obesity, an altered lipid profile (dyslipidaemia), and inflammation which increases the likelihood of developing atherosclerosis.

Carotid intima-media thickness (IMT) is a quantitative measure of subclinical atherosclerosis [1]. During the progression of atherosclerosis, there are arterial wall vessels changes characterized by a gradual increase in carotid IMT. Carotid IMT is one of the best established and most commonly used early surrogate markers of atherosclerosis [2] as well as a strong predictor of myocardial infarction [3]. Subclinical measurements of carotid IMT have been proposed to be good quantitative phenotypes for the evaluation of its genetic determinants [4]. It has been reported that there is an estimated heritability of 32%-59% in carotid IMT across different populations, indicating genetic factors play an important role in carotid IMT [5-7].

Endothelin-1 (ET-1), encoded by the EDN1 gene located in chromosome 6p21-24, is a potent vasoconstrictor in the body. ET-1 is expressed in several tissues, including endothelial cells and cardiomyocytes [8], and acts as a modulator of vasomotor tone and vascular remodeling [9,10]. ET-1 is present in atherosclerotic lesions, and mediates endothelial dysfunction by increasing fibroblast and macrophage activity [11]. ET-1 itself has been considered as a preclinical risk factor for cardiovascular disease [12,13]. In addition, ET-1 levels are associated with insulin resistance and impairment of glucose transport [14,16], and the one common polymorphism in the *EDN1* gene (rs5390 - Lys198Asn) has been reported to be associated to blood pressure in interaction with body mass index in European [17] and Japanese [18,19] populations. One important single-nucleotide polymorphism (SNP) [-1394TNG (rs1800541)] in the promoter region of EDN1 gene has been associated with a risk of developing myocardial infarction, rheumatoid arthritis, and asthma [20,24].

Endothelin type A receptor (EDNRA) is a receptor for ET-1, a potent vasoconstrictor. EDNRA is expressed in vascular smooth-muscle cells [25]. Two studies have investigated the association between single nucleotide polymorphisms (SNPs) in EDNRA and carotid IMT [26,27], but they showed conflicting results. One study identified that rs6841473 in the EDNRA gene modified the association between smoking and left carotid IMT in Africa Americans [26], whereas the other study did not find the association between rs1878406 in the EDNRA gene and carotid IMT in patients with rheumatoid arthritis [27].

The effects of the genetic loci of the *EDN1* *and* *EDNRA* genes in Han Chinese populations remain unclear. The paucity of research in this area highlights a need for additional study of the relation between endothelial system genes and carotid IMT. Furthermore, very few studies have focused on the interaction between these genes and environmental factors. Therefore, in our study we examined the associations of five SNPs in EDN1 and EDNRA genes with carotid IMT in a Han Chinese sample from the Taichung Community Health Study (TCHS), and evaluated their interaction effects by gender, regular exercise, and obesity status.

## 2. Methods

A community-based family study of subclinical atherosclerosis was conducted. All index subjects were identified from our cohort of Taichung Health Study (TCHS), conducted in 2004. TCHS consisted of 2,359 adults aged ≥40 years who were randomly selected from among Taichung’s general population. This cohort was longitudinally followed from October 2006 to 2009. A total of 1,666 residents were followed and the overall follow-up rate was 73.3%. Carotid IMT was measured at the second wave of the survey. This study was approved by the Human Research Committee of China Medical University Hospital. Informed consent was obtained from each participant.

All study subjects and their relatives were contacted individually by letter and phone and invited to participate in the study. Data were collected for these study subjects, their spouses, and their all first-degree blood relatives (parents, biological siblings, and offspring) who were 20 years of age or older. A total of 160 index subjects who could provide information on their spouse, and one first-degree blood relative of each index participant were included for this genetic study, for a total of 480 people. The inclusion criterion for study subjects was that they must have more than one first-degree relatives.

## 3. Measurements

### 3.1. Anthropometric measurement

Anthropometric measurements and blood samples were obtained from the complete physical examination. Weight and height were measured on an autoanthropometer (super-view, HW-666), with the subjects shoeless and wearing light clothing. Body mass index (BMI) was derived from the formula of weight (kg) ÷ (height)^2^ (m)^2^. Obesity was defined as a BMI greater than or equal to 27 kg/m^2^.

### 3.2. Extracranial carotid artery ultrasound measurement

After resting for at least 10 min in the supine position with the neck in slight hyperextension, all study subjects underwent a carotid ultrasound examination using a 7.5-MHz probe to scan the near and far wall of their arterial segments bilaterally in order to get the longitudinal (anterior oblique, lateral, and posterior oblique) and transverse views (GE L7000, GE, Milwaukee, Wis., U.S.A.). The protocol involved scanning the common carotid arteries (CCAs), the carotid bifurcations (bulb), and the origins (the first 1 cm) of the internal carotid arteries (ICAs). The near and far walls of these arterial segments were scanned longitudinally and transversally. The CCA image was taken just before the carotid artery bulb (≈5 mm) (CCA IMT). The carotid artery bulb was measured at the level of the proximal ICA sinus (bulb IMT), typically centered on the flow divider. The ICA measurement was made in the ICA where the walls were again parallel (ICA IMT).

### 3.3. Genetic analysis

Genomic DNA was isolated from the blood samples using the QIAamp DNA Blood Kit (Qiagen, Chatsworth, CA, USA). The concentration of the purified DNA was quantified using a ND- 2000c spectrophotometer (NanoDrop Technologies, Wilmington, DE, USA). We selected the rs1395821, rs1878406, and rs5333 SNPs of the EDNRA gene, and the rs1800541 and rs5370 of the EDN1 gene based on the findings for the CHB population in the International HapMap Project. The SNP genotyping was performed using an Illumina VeraCode GoldenGate genotyping assay (Illumina, San Diego, CA, USA).

### 3.4. Sociodemographic factors and life style behaviors

**Table 1 -:** Characteristics of study subjects.

		Total (n = 480)		Men (n = 251, 52.3%)		Women (n = 229, 47.7%)
Age (years)		51.39 ± 15.08		51.49 ± 16.28		51.28 ± 13.67
BMI (kg/m^2^)		23.93 ± 3.38		24.73 ± 3.41		23.05 ± 3.14
Obesity		77 (16.4)		53 (21.12)		24 (10.48)
**Health behavior**						
Regular exercise		306 (63.75)		165 (65.74)		141 (61.57)
Smoking		84 (17.5)		79(31.47)		5(2.18)
Drinking		93 (19.38)		76 (30.28)		17 (7.42)
Betel nut chewing		13 (2.71)		13 (5.18)		0 (0)
**Disease History**						
Hypertension		121 (25.21)		77 (30.68)		44 (19.21)
Hyperglycemia		50 (10.44)		33 (13.15)		17 (7.46)
Hyperlipidemia		162 (33.89)		87 (34.8)		75 (32.89)
CVA		6 (1.25)		4 (1.59)		2(0.87)
Cancer		18 (3.76)		6 (2.39)		12 (5.26)
IMT measurements						
IMT-CCA- distance (mm)		0.86 ± 0.29		0.91 ± 0.34		0.8 ± 0.22
IMT-Bulb- proximal (mm)		1.25 ± 0.70		1.31 ± 0.73		1.18 ± 0.66
IMT-ICA- proximal (mm)		0.86 ± 0.46		0.93 ± 0.51		0.79 ± 0.37

Data were presented as mean ± SD for continuous variables or n (%) for categorical variables. BMI: body mass index; CVA: cerebral vascular accident.

Data on sociodemographic characteristics, including gender, age, educational attainment, marital status, household income, smoking, drinking, physical activity, occupational activity, menopausal status, dietary habits, family history of cardiovascular-related diseases, physician-diagnosed diseases, and medication history were collected when the participants underwent a complete physical exam. Participants who engaged in regular exercise were defined as those who currently participated in regular leisure-time activities for at least 30 min per week for at least 6 months.

### 3.5. Statistical analysis

Simple descriptive analyses, such as mean, standard deviation, and proportion were presented. Generalized linear models (GLM) with generalized estimating equation(s) (GEE) were employed to compare differences in demographic factors, lifestyle behaviors, and disease history, including hypertension, hyperglycemia, hyperlipidemia, cardiovascular disease, and cancer among genotypes of SNPs in the EDNRA and EDN1 genes by considering the dependence among family members. Five SNPs were tested for deviation from HWE using PLINK software. Gene-environment interactions including gene-gender, gene-exercise, and gene-obesity interactions on carotid IMT were assessed by GLM with GEE adjusted for age, gender, obesity, regular exercise, smoking habits, alcohol drinking, and betel nut chewing. The gene-environment interactions were examined by entering the product terms of each environmental factor and individual SNP with either major al- lele as the reference group. In addition, three dummy variables measured the main effect of individual SNPs, the main effect of each environmental factor, and the combined effect of individual SNPs with each environmental factor. The values of carotid IMT for CCA, bulb, and ICA were natural log-transformed due to their skewed distributions. Adjusted mean ratio and 95% confidence intervals (CIs) of carotid IMT values were presented. Furthermore, the haplotypes in this sample were analyzed separately for each gene at a frequency > 5%. The significance level was set at a two-sided *P* < 0.05. All analyses used Statistical Analysis System software (v9.4, SAS Institute Inc., Cary, NC, USA), PLINK (v1.07) (http://pngu.mgh.harvard.edu/purcell/plink) [28], and Haploview (v4.2) [29].

## 4. Results

The characteristics of all study subjects and stratified by gender are shown in Table 1. The mean age of the community-dwelling men and women was 51.49 years (SD = 16.28 years) and 51.28 years (13.67 years), respectively. Among the study subjects, 52.3% were men. The prevalence of obesity, regular exercise, smoking, drinking, and betel nut chewing were 16.4%, 63.75%, 17.5%, 19.38%, and 2.71%, respectively. The self-reported diagnosed diseases included hypertension (25.21%), hyperglycemia (10.44%), hyperlipidemia (33.89%), CVA (1.25%), and cancer (3.76%). The mean values of CCA IMT, bulb IMT, and ICA IMT in men were 0.91 (0.34), 1.31 (0.73), and 0.93 (0.51), respectively; and in women were 0.8 (0.22), 1.18 (0.66), and 0.79 (0.37), respectively.

**Table 2 −:** Genotype distributions of study subjects and IMT distributions according to genotype status.

Gene		SNP		Genotype or allele		n (%)		CCA IMT ^#^		BULB IMT ^#^		ICA IMT ^#^
EDNRA		rs1395821		A A		184 (38.33)		0.84 ± 1.35		1.13 ± 1.63		0.82 ± 1.52
				A G		229 (47.71)		0.82 ± 1.33		1.11 ± 1.62		0.78 ± 1.45
				G G		64 (13.33)		0.78 ± 1.29		1.02 ± 1.57		0.74 ± 1.41
				G*		357 (0.37)						
EDNRA		rs1878406		A A		22 (4.58)		0.84 ± 1.37		1.2 ± 1.7		0.89 ± 1.52
				A G		158 (32.92)		0.83 ± 1.33		1.15 ± 1.63		0.8 ± 1.48
				G G		298 (62.08)		0.81 ± 1.33		1.08 ± 1.61		0.78 ± 1.47
				A*		202 (0.27)						
EDNRA		rs5333		A A		300 (62.5)		0.82 ± 1.34		1.09 ± 1.62		0.78 ± 1.47
				A G		161 (33.54)		0.83 ± 1.32		1.14 ± 1.63		0.79 ± 1.48
				G G		18 (3.75)		0.8 ± 1.27		1 ± 1.47		0.85 ± 1.5
				G*		197 (0.21)						
EDN1		rs1800541		A A		313 (65.21)		0.81 ± 1.31		1.09 ± 1.61		0.78 ± 1.48
				A C		148 (30.83)		0.84 ± 1.36		1.14 ± 1.65		0.81 ± 1.46
				C C		18 (3.75)		0.8 ± 1.36		1.06 ± 1.42		0.73 ± 1.43
				C*		184 (0.19)						
EDN1		rs5370		A A		34 (7.08)		0.82 ± 1.32		1.11 ± 1.62		0.78 ± 1.47
				A C		191 (39.79)		0.83 ± 1.34		1.11 ± 1.63		0.8 ± 1.47
				C C		250 (52.08)		0.8 ± 1.37		1.04 ± 1.56		0.81 ± 1.5
				A*		259 (0.37)						

Data were presented as n (%) for genotypes and alleles or mean ± SD for IMT.

#: Geometric mean was presented.

*: Minor allele.

**Table 3 −:** Significant gene-environment interactions on CCA-distance, BULB-proximal, and ICAproximal.

Environment		Gene		SNP		P for interaction				
						CCA IMT		BULB IMT		ICA IMT
Gender										
		EDNRA		rs1395821		0.03^d^				0.026^d^
		EDNRA		rs1878406		0.017^r^				
		EDNRA		rs5333		0.036 ^r^				
		EDN1		rs1800541				0.024 ^d^		
		EDN1		rs5370				0.019 ^d^		0.033^r^
Regular exercise										
		EDNRA		rs1395821				0.02 ^r^		0.031^r^
		EDNRA		rs5333				0.002 ^r^		
Obesity										
		EDNRA		rs5333		0.027 ^r^				

d: interaction term was assessed by a dominant model; r: interaction term was assessed by a recessive model.

All five of the SNPs studied in the two genes were within the HWE (*P* > 0.05) and the allele and genotype distributions for all polymorphisms are shown in Table 2. The associations of the EDNRA and EDN1 polymorphisms with CCA IMT, bulb IMT, and ICA IMT were examined. The mean values of CCA IMT, bulb IMT, and ICA IMT were not significantly different among genotypes of these 5 SNPs (data not shown). Multiple linear regression analyses were performed on haplotypes in the EDN1 and EDNRA genes. The haplotypes with a frequency of >5% were retained for analysis and we identified four haplotypes of the EDNRA gene for analysis. Two haplotypes of the EDNRA gene were prevalent (A-G-A: 45% and G-G-A: 26.7%), and the other two haplotypes were less common with estimated frequencies of 8.34% and 5.02%. Individuals who carried the G-G-A EDNRA haplotype displayed significantly thinner ICA IMT without adjustment (ß = -0.07 [95% CI: -0.13, -0.01]; *P* < 0.05). After multivariate adjustment, this effect became borderline significant.

The gene-gender, gene-exercise, and gene-obesity interactions for EDNRA and EDN1 SNPs on carotid IMT were explored (Table 3). After multivariate adjustment, the effects of interactions between EDNRA SNPs rs1395821, rs1878406, and rs5333 with gender as well as EDNRA SNP rs5333 with obesity on CCA IMT were observed. We also observed significant interactions of EDN1 SNPs rs1800541 and rs5370 with gender as well as EDNRA SNPs rs1395821 and rs5333 with regular exercise on bulb IMT. As for ICA IMT, the interactions of EDNRA SNP rs1395821 and EDN1 SNP rs5370 with gender, as well as EDNRA SNP rs1395821 with regular exercise were significant.

**Fig. 1 –:**
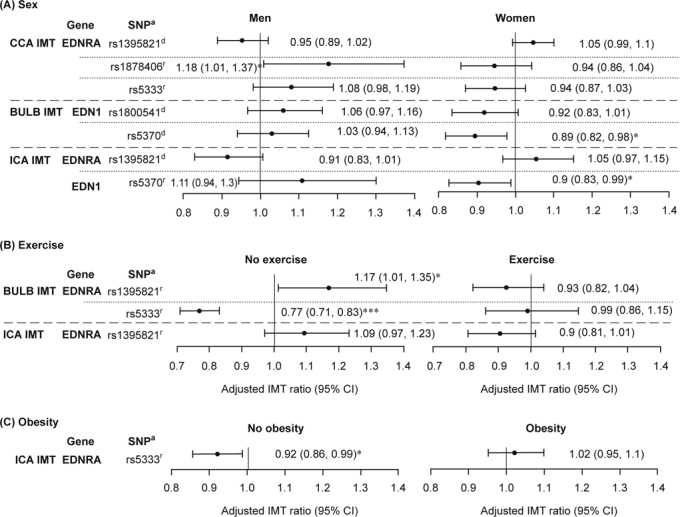
Stratified analysis by gender (A), exercise (B), and obesity (C) for significant interaction of *EDNRA* rs1395821, rs1878406, and rs5333, as well as EDN1 rs1800541, and rs5370 with gender, exercise, and obesity on CCA, bulb, and ICA IMTs are shown. The adjusted mean ratios were obtained from the models with consideration of SNP, age, gender, obesity, smoking, alcohol drinking, and betel nut chewing. *P 003C; 0.05. r: recessive model; d: dominant model.

To explore the significant interactions identified above, we estimated the adjusted mean ratio in carotid IMT measurements for either EDNRA or EDN1 minor—major/minor—minor genotypes in comparison with the major—major genotype stratified by gender, regular exercise, and obesity (Figure 1). For gene-gender interaction on CCA IMT, the adjusted mean for men carrying the GA/GG genotype of EDNRASNP rs1878406 was 1.18 times higher than that for men carrying the AA genotype (95% CI: 1.01, 1.37). On the other hand, we did not observe significant effects for ENDRA SNP rs1878406 in women. As for bulb and ICA IMT, the adjusted mean values for women carrying the AC/AA genotype of EDN1 rs5370 was lower than those carrying the CC genotype (0.89, [0.82, 0.98]; and 0.9 [0.83, 0.99], respectively). But the effects of EDN1 rs5370 on bulb and ICA IMT were not significant in men (Figure 1A). For gene-exercise interaction, we observed that individuals carrying the GA/GG genotype of EDNRA SNP rs1395821 had higher mean values of bulb IMT than those carrying the AA genotype among individuals who did not regular exercise. Whereas individuals carrying the GA/GG genotype of EDNRA SNP rs5333 had higher mean values of bulb IMT than those carrying the AA genotype among the subjects who did not regularly exercise. On the other hand, we did not observe significant effects of EDNRA SNPs rs1395821 and rs5333 in individuals who regularly exercised (Figure 1B). A significantly lower adjusted mean in CCA IMT for non-obese individuals carrying EDNRA SNP rs5333 was observed (0.92 [0.86, 0.99]) compared with non-obese individuals carrying the AA genotype (Figure 1C). In contrast, it was found that EDNRA SNP rs5333 had no no effect on CCA IMT in obese individuals.

## 5. Discussion

ET-1 is a potent vasoconstrictor in the body and is involved with the biology of the vascular endothelium. In the present study, we examined the evidence for gene-gender, gene-exercise, and gene- obesity interaction between the two SNPs in the *EDN1* gene and three SNPs in the EDNRA gene in relation to carotid IMT in Han Chinese subjects of Taiwan. We found gender, regular exercise, and obesity significantly interacted with SNPs in the EDNRA gene, and gender interacted with SNPs in the EDN1 gene to influence carotid IMTs in the Han Chinese participants of the TCHS study. Stratified analysis indicated that EDNRA SNP rs1878406 was associated with CCA IMT in male participants, while EDN1 SNP rs5333 was associated with bulb and ICA IMT in women. For EDNRA SNP rs1878406, the minor allele (A) was associated with higher CCA IMT levels in men whereas the minor allele (G) of EDN1 SNP rs5333 was associated with lower bulb and ICA IMT levels in women. Furthermore, the minor allele (G) of EDNRA SNP rs5333 decreased bulb and CCA IMT levels in individuals without regular exercise or obesity whereas the minor allele (G) of EDNRA SNP rs1395821 increased bulb IMT level in individuals without regular exercise. These findings could provide valuable information towards delineating the genetic mechanisms underlying carotid IMTs.

Previous epidemiological genetic studies of ET-1 have principally focused on hypertensive properties [17-19, 30], as have genetic studies of EDNRA [31]. Few studies thus far have explored their associations with IMT [26, 27, 30, 32]. One prior study explored the interaction of smoking with SNP rs6841473 in EDNRA on left carotid IMT in Africa Americans. The minor allele (T) of rs6841473 increased the left carotid IMT level in non-smoking Africa Americans, but had little effect in black smokers [26]. Another study examined the association between mean carotid intima media thickness and EDN1 SNP rs5370 in a general population sample of Western Australia and they found the minor allele was marginally associated with increased mean IMT levels [30]. Another study explored the potential relationship between *EDNRA* rs1878406 polymorphisms and carotid IMT in patients with rheumatoid arthritis, and no statistically significant differences were found when this polymorphism was assessed according to carotid IMT values [27]. A study conducted in Japanese patients with essential hypertension investigated the relationship between 11 SNPs of ET-1 family genes (including three in EDN1, one in EDNRA) and atherosclerotic changes and found a significant correlation between mean carotid IMT and EDNRArs5333 in male, but not female, hypertensive patients [32]. In the present study, we did not observe main effects of rs1395821, rs1878406, and rs5333 of the EDNRA gene, and rs1800541, and rs5370 of the EDN1 gene on carotid IMT levels. Instead, we identified a significant interaction of gender with these three SNPs in the EDNRA gene and with these two SNPs in the EDN1 gene, regular exercise with rs1395821 and rs5333 in the EDNRA gene, and obesity with rs5333 in the EDNRA gene in Han Chinese subjects. When we stratified our study sample by gender, we observed no significant evidence for associations between some SNPs and carotid IMT in each of the two strata due to a limited sample size in each stratum.

EDNRA encodes the receptor for ET-1, which plays an important role in potent and long-lasting vasoconstriction [26,33]. Endothelium dysfunction is accompanied in atherosclerosis [26,33]. Previous bench research found that endothelin receptor antagonists are effective in attenuating vascular abnormality in atherosclerosis [26,33]. As for EDN1, it encodes preproendothelin that is processed to form endothelin 1, a potent vasoconstrictor. SNP rs1800541 is located in the EDN1 promoter region with potential regulatory effects on gene expression and rs5370 is one common non-synonymous SNP that is located in the CT-pro- ET-1 part of the ET-1 coding gene. Previous studies have reported rs5370 to be associated with idiopathic pulmonary arterial hypertension [34] and risk for hypertension [10]. The potential physiological functions of these two genes, along with the significant associations in our study and the previous studies [26,30], implicate them as strong candidate genes for atherosclerosis. Never- theless, future functional studies in humans may be necessary.

One limitation of the present study is that the sample size of our study was relatively small, particularly in the evaluation of gene-gender interactions. Therefore, we may have missed important polymorphisms that are associated with carotid IMT due to a low statistical power for stratified analysis. Future studies with a larger number of participants are needed.

Our study had important strengths. First, we assessed for the first time the potential association between the 5 polymorphisms in EDNRA and EDN1 genes and subclinical atherosclerosis as well as the interactions between these 5 variants with gender, regular exercise, and obesity in Han Chinese. Second, our study subjects were from a representative sample of Taiwan's Chinese population. Therefore, the generalization of our results to other Han Chinese populations is applicable. Third, the measurement of both carotid IMT and genotypes had stringent quality controls.

In conclusion, the current study identified three variants (rs1395821, rs1878406, rs5333) in the EDNRA gene and two variants (rs1800541 and rs5370) in the EDN1 gene that interacted with gender; two in the EDNRA gene (rs1395821 and rs5333) interacted with regular exercise; and one in the EDNRA gene (rs5333) interacted with obesity on carotid IMT in Han Chinese participants from the TCHS. These findings help delineate the genomic mechanisms underlying carotid IMT. Still, further studies will be necessary to genotype other important variants underlying the EDNRA and EDN1 signals, and to elucidate the mechanisms with which gender, regular exercise, and obesity modify the effects of these polymorphisms on carotid IMT in Han Chinese.

## Acknowledgments

This study is supported in part by the Ministry of Science and Technology of Taiwan (National Science Council) (NSC 99-2628- B-039-007-MY3 & NSC 94-2314-B-039-019), the Taiwan Ministry of Health and Welfare Clinical Trial and Research Center of Excellence (M0HW104-TDU-B-212-113002), and the China Medical University (CMU102-BC-11).

## Declaration of Interest

Authors declare no conflicts of interest for this work.
